# U.S. cereal rye winter cover crop growth database

**DOI:** 10.1038/s41597-024-02996-9

**Published:** 2024-02-13

**Authors:** Alexandra M. Huddell, Resham Thapa, Guillermo S. Marcillo, Lori J. Abendroth, Victoria J. Ackroyd, Shalamar D. Armstrong, Gautam Asmita, Muthukumar V. Bagavathiannan, Kipling S. Balkcom, Andrea Basche, Shawn Beam, Kevin Bradley, Lucas Pecci Canisares, Heather Darby, Adam S. Davis, Pratap Devkota, Warren A. Dick, Jeffery A. Evans, Wesley J. Everman, Tauana Ferreira de Almeida, Michael L. Flessner, Lisa M. Fultz, Stefan Gailans, Masoud Hashemi, Joseph Haymaker, Matthew J. Helmers, Nicholas Jordan, Thomas C. Kaspar, Quirine M. Ketterings, Eileen Kladivko, Alexandra Kravchenko, Eugene P. Law, Lauren Lazaro, Ramon G. Leon, Jeffrey Liebert, John Lindquist, Kristen Loria, Jodie M. McVane, Jarrod O. Miller, Michael J. Mulvaney, Nsalambi V. Nkongolo, Jason K. Norsworthy, Binaya Parajuli, Christopher Pelzer, Cara Peterson, Hanna Poffenbarger, Pratima Poudel, Mark S. Reiter, Matt Ruark, Matthew R. Ryan, Spencer Samuelson, John E. Sawyer, Sarah Seehaver, Lovreet S. Shergill, Yogendra Raj Upadhyaya, Mark VanGessel, Ashley L. Waggoner, John M. Wallace, Samantha Wells, Charles White, Bethany Wolters, Alex Woodley, Rongzhong Ye, Eric Youngerman, Brian A. Needelman, Steven B. Mirsky

**Affiliations:** 1 Department of Environmental Science & Technology, University of Maryland, College Park, MD USA; 2 Department of Plant and Soil Sciences, University of Delaware, Newark, DE USA; 3 Department of Agricultural and Environmental Sciences, Tennessee State University, Nashville, TN USA; 4 Department of Agricultural Sciences, West Texas A&M University, Canyon, TX USA; 5 USDA-ARS, Cropping Systems and Water Quality Research Unit, Columbia, MO USA; 6 Department of Plant Science & Landscape Architecture, University of Maryland, College Park, MD USA; 7 Department of Agronomy, Purdue University, West Lafayette, IN USA; 8 Department of Soil and Crop Sciences, Texas A&M University, College Station, TX USA; 9 USDA-ARS, National Soil Dynamics Laboratory, Auburn, AL USA; 10 Department of Agronomy and Horticulture, University of Nebraska-Lincoln, Lincoln, NE USA; 11 School of Plant and Environmental Sciences, Virginia Tech, Blacksburg, VA USA; 12 University of Missouri, Columbia, MO USA; 13 Department of Plant and Soil Sciences, University of Kentucky, Lexington, KY USA; 14 University of Vermont Extension, St. Albans, VT USA; 15 USDA-ARS, Global Change and Photosynthesis Research Unit, Urbana, IL USA; 16 Department of Crop Sciences, University of Illinois, Urbana, IL USA; 17 West Florida Research and Education Center, University of Florida, Institute of Food and Agricultural Sciences, Jay, FL USA; 18 School of Environment and Resources, Ohio State University, Wooster, OH USA; 19 Farmscape Analytics, Concord, NH USA; 20 Department of Crop and Soil Sciences, North Carolina State University, Raleigh, NC USA; 21 School of Plant, Environmental and Soil Sciences, Louisiana State University AgCenter, Baton Rouge, LA USA; 22 Practical Farmers of Iowa, Ames, IA USA; 23 Stockbridge School of Agriculture, University of Massachusetts Amherst, Amherst, MA USA; 24 Eastern Shore Agricultural Research and Extension Center, Virginia Tech, Painter, VA USA; 25 Iowa Nutrient Research Center, Department of Agriculture and Biosystems Engineering, Iowa State University, Ames, IA USA; 26 Agronomy and Plant Genetics Department, University of Minnesota, St. Paul, MN USA; 27 USDA-ARS, National Laboratory for Agriculture and the Environment, Ames, IA USA; 28 Nutrient Management Spear Program, Department of Animal Science, Cornell University, Ithaca, NY USA; 29 Department of Plant, Soil and Microbial Sciences, Michigan State University, East Lansing, MI USA; 30 Blue River Technology, and Lousiana State University AgCenter, Baton Rouge, LA USA; 31 Soil and Crop Sciences Section, School of Integrative Plant Science, Cornell University, Ithaca, NY US; 32 Department of Natural Resource Sciences, McGill University, Montreal, Quebec Canada; 33 Carvel Research and Education Center, University of Delaware, Georgetown, DE USA; 34 Department of Plant & Soil Sciences, Mississippi State University, Starkville, MS USA; 35 Navajo Technical University, Crownpoint, NM USA; 36 University of Arkansas Systems Division of Agriculture, Little Rock, Arkansas USA; 37 Department of Plant & Environmental Sciences, Clemson University, Florence, SC USA; 38 Department of Soil Science, University of Wisconsin–Madison, Madison, WI USA; 39 Department of Agronomy, Iowa State University, Ames, IA USA; 40 Southern Ag Research Center, Montana State University, Huntley, MT USA; 41 Department of Plant Science, Pennsylvania State University, University Park, PA USA; 42 Department of Agriculture, Geosciences and Natural Resources, University of Tennessee at Martin, Martin, TN, USA; 43 U.S. Department of Agriculture, Agricultural Research Service, Sustainable Agricultural Systems Laboratory, Beltsville Agricultural Research Station, Beltsville, MD USA

**Keywords:** Agroecology, Plant physiology

## Abstract

Winter cover crop performance metrics (i.e., vegetative biomass quantity and quality) affect ecosystem services provisions, but they vary widely due to differences in agronomic practices, soil properties, and climate. Cereal rye (S*ecale cereale*) is the most common winter cover crop in the United States due to its winter hardiness, low seed cost, and high biomass production. We compiled data on cereal rye winter cover crop performance metrics, agronomic practices, and soil properties across the eastern half of the United States. The dataset includes a total of 5,695 cereal rye biomass observations across 208 site-years between 2001–2022 and encompasses a wide range of agronomic, soils, and climate conditions. Cereal rye biomass values had a mean of 3,428 kg ha^−1^, a median of 2,458 kg ha^−1^, and a standard deviation of 3,163 kg ha^−1^. The data can be used for empirical analyses, to calibrate, validate, and evaluate process-based models, and to develop decision support tools for management and policy decisions.

## Background & Summary

Winter cover crops provide many ecosystem services such as weed suppression, improved soil structural and hydraulic properties, increased soil organic carbon (C) stocks, reduced erosion, reduced winter nitrogen (N) leaching, and N provision to cash crops^1–4^. Cover crop biomass production is often positively correlated to ecosystem service provisions^1^. For example, previous research has shown that weed biomass often decreases with greater cereal rye (S*ecale cereale*) residue^5,6^, and soil organic C often increases with cover crop biomass^7,8^. Similarly, there were greater reductions in nitrate leaching with increases in non-leguminous cover crop shoot biomass in a global meta-analysis^3^. Elucidating the agronomic, soils, and climate controls on winter cover crop performance can help farmers determine the optimal time to terminate cover crops for maximum agronomic benefits, and support broader adoption of winter cover crops for increased climate resilience, reduced soil erosion, and lower nutrient pollution^2^.

**Fig. 1:**
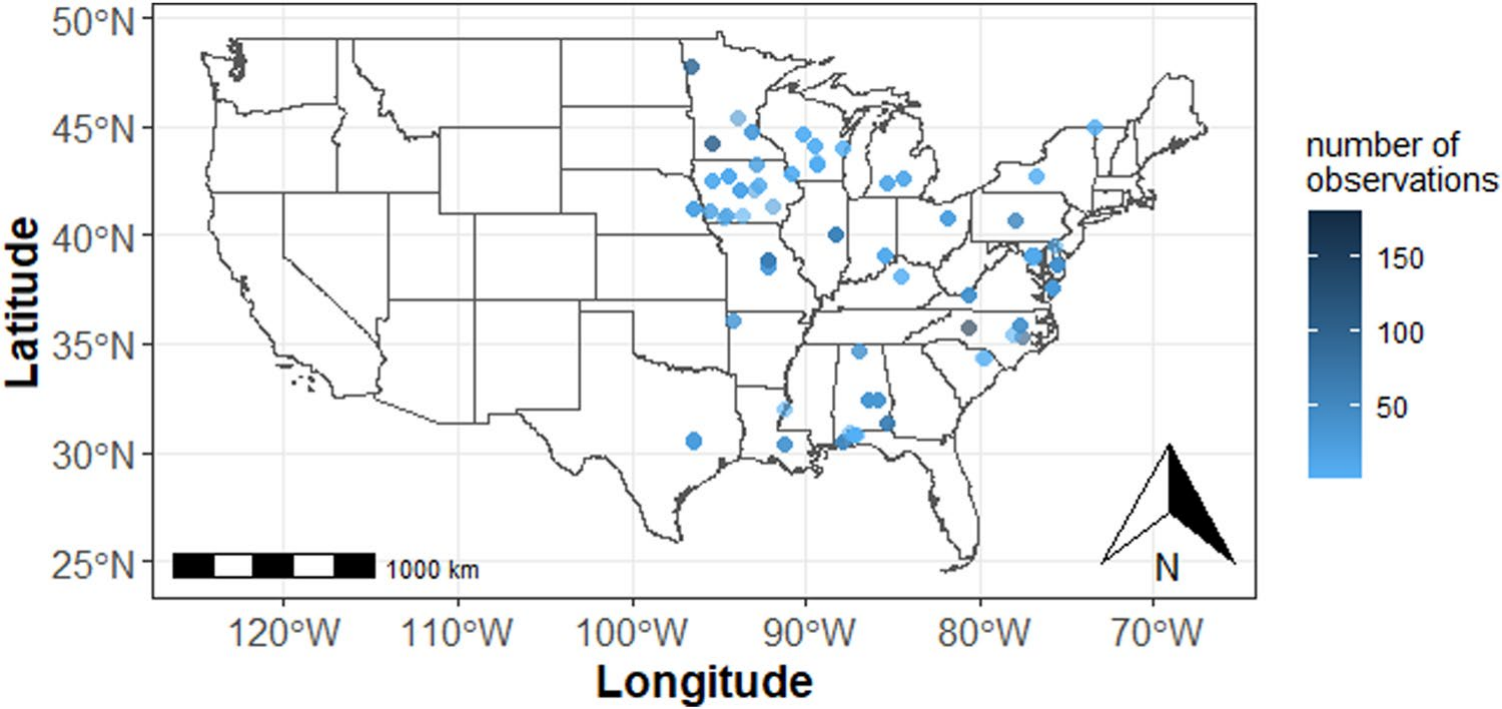
Map of research locations with the point color scaled by the number of available observations.

We focused on performance data of cereal rye, the most commonly used cover crops in the United States^9,10^. To acquire data for this study, we reached out to potential data contributors through regional cover crop groups and the Precision Sustainable Agriculture and Getting Rid of Weeds networks, two national research consortia with a major focus on cover crop research. Recruiting data contributors through this network enabled us to assimilate many plot-level observations from both on-station and on-farm studies (Fig. 1). Our goal was to create a dataset on cereal rye cover crop biomass quantity and quality across heterogeneous agronomic, soils, and climate conditions with broad coverage across the eastern half of United States (Fig. 1).

## Methods

We collected data on cereal rye cover crop performance metrics (biomass, N content, and C:N ratio), additional agronomic and soil data, and metadata such as any associated publications (Table 1). The minimum data required from a location for inclusion in our dataset was aboveground (shoot) cereal rye biomass, the respective harvest or sampling date, the experimental site name, year, latitude and longitude, cereal rye planting date, cereal rye planting method (drilled vs. broadcast), and whether N fertilizer was applied during cover crop growth (Table 1).

**Table 1:** Structure of the data records with file name, purpose, and variables included for each tabular dataset included.

File description	File name	Purpose	Variables included
Data dictionary	1_data_dictionary.csv	Definition of variables for the following files	**File name, attribute name, attribute definition**, string format, unit, number type
Study metadata	2_study_metadata.csv	Detailed site location and study design information	**study ID, site ID, year, state, latitude, longitude, whether location was obscured for privacy, experimental design, number of replications, publication DOI, methods for unpublished data**
Rye data	3_rye_data.csv	Cereal rye related data associated with biomass observations	**study ID, site ID, year, planting and sampling dates**, N rate applied fall or spring to cover crop, cultivar, **biomass**, C and N data, seeding rate, cumulative growing degree days, plant population measurements
Agronomic data	4_agronomic_data.csv	Agronomic management data general to each study	**study ID, site ID, year**, previous and next cash crop, **planting method**, fall tillage, row spacing, **whether N fertilizer was applied during cover crop growth**, N fertilization date
Soil data	5_soil_data.csv	Soil sample data and/or general site soils data	**study ID, site ID, year, whether soil samples were taken**, sampling event timing and depths, soil ammonium, soil nitrate, total inorganic N, soil texture class, sand %, silt %, clay %, bulk density, percent soil organic matter or carbon, pH

Bolded variables were required for inclusion in the (minimum) dataset; other variables were not available for all sites.

Additional data gathered included cereal rye cultivar; seeding rate; row spacing; plant population; tiller density; growth stage at sampling; cumulative growing degree days; shoot N (concentration or content); shoot C:N ratio; N fertilizer rate, type (form), and application date; presence of fall tillage; and previous and subsequent cash crop. Root C and N data were requested but not available for any study. Additional soil data gathered included texture class and/or clay, silt, and sand percentages; bulk density; soil pH; soil ammonium, nitrate, and/or total inorganic N; soil organic matter or C; as well as soil sampling depth and timing. The overwhelming majority of observations were recorded as plot level data. In the few cases (17 observations) when plot level data were not available, we collected treatment means with standard deviations (Table 1). At least 14 of the 28 studies correspond to existing publications, which are detailed in the study metadata CSV file^11–25^. Plot sizes varied from as small as 6.1 × 6.1 m to as large as 30.5 × 42.7 m depending on the study. These published datasets described methods for cereal rye biomass collection and some soil analyses in each publication. The DOI from any publications associated with data provided is listed in the “publication_DOI” column in the study metadata file, and “NA” is listed for studies with unpublished data. The methods for cereal rye biomass collection and some soil analyses and unpublished are detailed in the “methods_unpublished_data” column of the study metadata file.

To focus on the vegetative biomass of cereal rye that is planted in the fall and followed by cash crops in the spring, we only included biomass sampling that occurred in February, March, April, or May. We excluded observations from sites that terminated winter cover crops after May—such as forage experiments where cover crop vegetative biomass and grain were sampled in June or later. We also omitted biomass data outliers that fell above the 99.9^th^ percentile (n = 6) because the upper end of the distribution appeared to have unrealistic values. All figures and summary statistics were created using the statistical software *R* (v4.1.3)^26^ and the following packages: *ggplot2* v3.4.1^27^, *ggmap* v3.0.1^28^, and *ggsn* 0.5.0^29^.

## Data Records

The metadata and primary data collected can be accessed through the following repository: 10.5061/dryad.tx95x6b3h^30^. The data are organized in tabular CSV files including a data dictionary, study metadata, rye data, agronomic data, and soil data. (Table 1). In 26 out of 245 site years, location data from private farms were obscured to ensure privacy and have lower location accuracy than the rest of the dataset; as such, those locations are indicated as “TRUE” in the “location_obscured” variable. We reported location data with as much precision as possible for each datapoint, as a result, there are varying levels of precision in reported latitude and longitude coordinates.

## Technical Validation

**Fig. 2:**
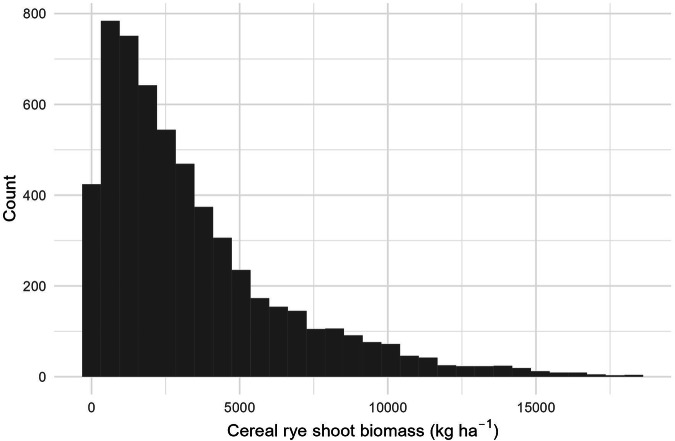
Histogram of cereal rye shoot dry biomass data.

**Fig. 3:**
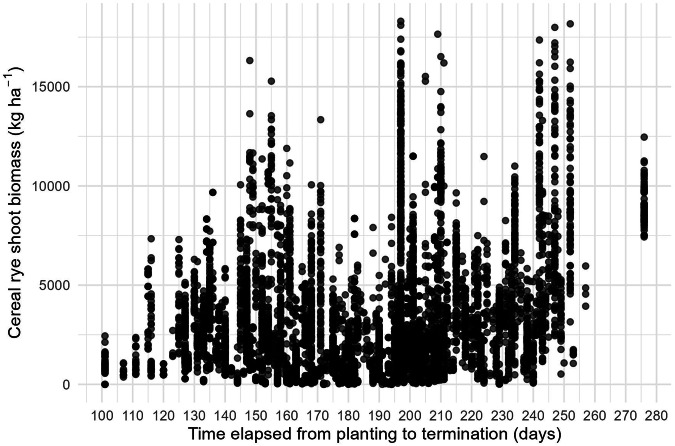
Cereal rye shoot biomass versus time elapsed between cereal rye planting to termination date.

**Fig. 4:**
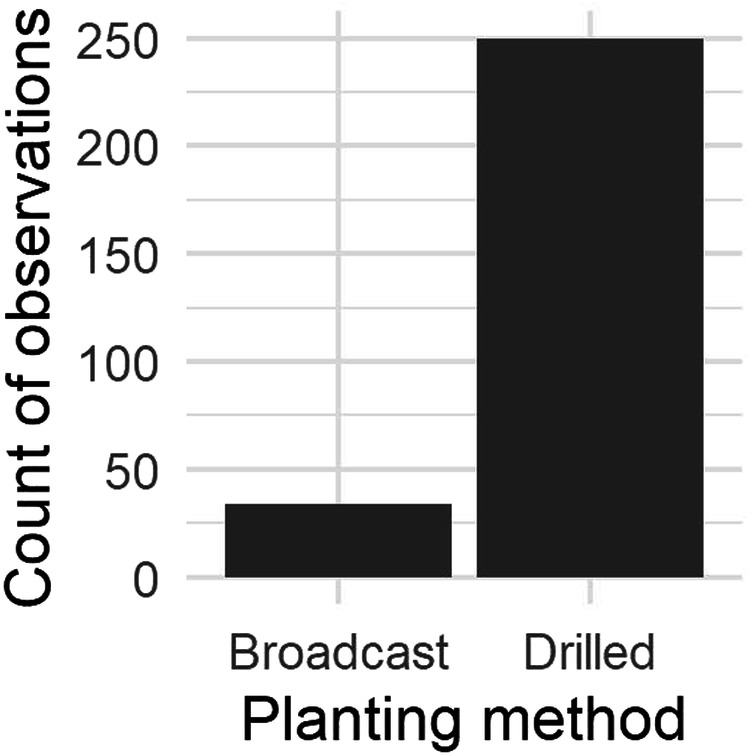
Summary of planting method data.

To check the validity of the data we collected, we examined the spread of the data and found some unreasonable values. Cereal rye biomass data outliers that fell above the 99.9^th^ percentile were excluded. The final dataset had a mean of 3,428 kg ha^−1^, a median of 2,458 kg ha^−1^ and a standard deviation of 3,163 kg ha^−1^ (Fig. 2). We also checked overall data validity by assessing whether cereal rye biomass generally increased with time elapsed from planting to termination date. This increase was observed; however, there was a large amount of variability (Fig. 3). This variability in cereal rye biomass production may be explained by differences in agronomic, soil, and climate factors. A subset of commonly reported ancillary data is summarized in Table 2 and Fig. 4.

**Table 2:** Summary of commonly-reported ancillary data from the rye growth dataset.

Variable	Mean	Standard deviation	Observations with reported data (%)
Fall fertilization rate (kg N ha^−1^)	4.7	14.7	93.0
Spring fertilization rate (kg N ha^−1^)	17.4	37.0	93.0
Shoot N (%)	2.5	5.23	40.8
Shoot C:N	32.0	22.5	38.3
Seeding rate (kg ha^−1^)	101	35.3	50.4

## Data Availability

The code used to create figures for this study can be accessed at the following repository: 10.5061/dryad.tx95x6b3h^30^. Figures and analyses were produced using *R* v4.1.3.
